# Job-demand and family business resources in pandemic context: How they influence burnout and job satisfaction

**DOI:** 10.3389/fpsyg.2022.1061612

**Published:** 2023-01-05

**Authors:** Orlando Llanos-Contreras, María José Ibáñez, Vicente Javier Prado-Gascó

**Affiliations:** ^1^Facultad de Ciencias Económicas y Administrativas, Universidad Católica de la Santísima Concepción, Concepción, Chile; ^2^CENTRUM Católica Graduate Business School, Lima, Peru; ^3^Pontificia Universidad Católica del Perú, Lima, Peru; ^4^Department of Social Psychology, Faculty of Psychology, University of Valencia, Valencia, Spain

**Keywords:** job satisfaction, burnout—professional, job demand-resources (JD-R) model, family firm, COVID-19

## Abstract

This research aims to explore how work demands and resource variables affect the burnout and satisfaction of employees of family businesses in the context of the pandemic (COVID-19) and the moderation effect of fear of COVID-19 on this relationship. A sample of 214 Chilean family business employees is used for hypotheses testing. Results indicate that the demands and resources partially explain the burnout and satisfaction of employees of family firms during the pandemic. Employees’ fear of COVID-19 moderates the relationship between resources-demands and burnout-job satisfaction in family firms. This work contributes to understanding how these organizations can manage adverse scenarios to survive and continue operations.

## 1. Introduction

Job satisfaction and burnout are two related variables that inform about employees’ psychosocial risk and occupational health in the workplace (Giménez-Espert et al., 2020). The most acknowledged models to assess organizational health based on these two variables are the job demand-control-support model (JDCS) and job demand-resource (JD-R) (Karasek and Theorell, 1990; Demerouti et al., 2001). These models proposed that the psychological work environment is defined by a combination of job demands, job control, and social support (Baka, 2020). In the assessment of the organizations’ psychosocial risk, these variables have been mostly assessed in relation to burnout and job satisfaction, proving their predictive ability (Pelfrene et al., 2001; Jalilian et al., 2019; Dutheil et al., 2020; Ellison and Caudill, 2020; Fernemark et al., 2020). Although the available research on this type of variables, as well as this reference model seems to be scarce in the context of family businesses. Recently, JD-R has been successfully used to predict job satisfaction among entrepreneurs running their own family business (McDowell et al., 2019).

The impact of the so-called psychosocial risks on the health of workers and, by extension, the productivity of organizations can be affected by external shocks, such as the case of the health emergency and its social, economic, and political consequences caused by the COVID-19 pandemic (Prado-Gascó et al., 2020).

The COVID-19 pandemic has caused an unprecedented economic impact worldwide due to the closure of businesses, the cessation of any non-essential activity, and the reduction of mobility during the different confinements, which has led to the closure of many companies and a large destruction of employment (Apedo-Amah et al., 2020).

De Massis and Rondi (2020) have argued that the COVID-19 pandemic has triggered challenges for family business management and research. One area in which this assumption is questioned is how emotions and stress resulting from the pandemic pressure influence these firms’ behaviors and ability to survive. Recent research on the family businesses’ ability to face external disruptions has suggested that the priority for preserving socioemotional wealth is an engine which supports these businesses’ continuity when facing adversities (Alonso-Dos-Santos and Llanos-Contreras, 2019; Llanos-Contreras et al., 2020). Socioemotional wealth also enhances these firms’ concern for binding social ties with external and internal stakeholders, such as their employees (Berrone et al., 2012; Llanos-Contreras and Alonso-Dos-Santos, 2018). For this reason, it is expected that, despite the adverse consequences of the pandemic, these organizations would be concerned about keeping their worker’s occupational health under control. Hence, understanding the factors behind this problem is important for family firms, particularly in a pandemic which increases psychological risk at work and in parallel places the business’ continuity at risk.

In this context, the JD-R model has proven to be reliable even under highly stressful scenarios as the COVID-19 pandemic (e.g., Prado-Gascó et al., 2020; Jamal et al., 2021). However, it has never been tested for the assessment of employees’ satisfaction and burnout in family firms when these are facing adverse scenarios. There is research studying family firms’ post-disaster performance and behaviors (Marshall et al., 2015; Alonso-Dos-Santos and Llanos-Contreras, 2019; Salvato et al., 2020; Mahto et al., 2022). These studies acknowledged these firms’ abilities to adapt to challenging conditions, take risks, and leverage all their available resources to survive. But the focus has been mostly on general management issues at the family and business level, and no attention has been paid to employees’ responses toward external shock which increases the psychosocial risk.

For this reason, in this research, we aim to respond to the questions of how demands and resources variables affect the level of burnout and satisfaction of employees of family businesses in the context of the pandemic (COVID-19) and how fear of COVID-19 affects the influence of demands and resources variables on the level of burnout and employee satisfaction in family firms.

To answer these questions, a partial least squares structural equation analysis (SEM-PLS) was implemented using variables of organizational support, workload, and job insecurity to explain burnout and satisfaction in 214 employees of Chilean family businesses. Data were collected at the peak of the pandemic in Chile in terms of deaths and sanitary restrictions. To determine how fear of COVID-19 affects the relationships in the model, a multigroup analysis (MGA) was performed by grouping individuals into three categories: high, medium, and low, according to their level of fear of the pandemic. The results suggest that the demands and resources model partially support the predictions. Workers’ fear of COVID-19 was also found to have a moderating influence on the relationship between resources-demands and burnout-job satisfaction in family firms.

In this way, we contribute to family firm theory by making progress in the understanding of how these organizations can manage adverse scenarios to survive and continue operations (Marshall and Schrank, 2020; Mahto et al., 2022). Particularly, this research focused on determining job resources and demand that need to be correctly managed in order to keep psychological risk at work under control. In this way, we also contribute to the literature on organizational psychology (Bennis et al., 1966; Beehr, 1998), particularly on the study of psychosocial risk, by testing the prediction ability of the JD-R model in the particular context of family firms and the pandemic. Finally, we contribute to the very scant research in family firms in Latin America, a region where these organizations are particularly prevalent (Gomez-Mejia et al., 2020; Vazquez et al., 2020).

This article is organized as follows: The next section provides a discussion of the theoretical framework supporting the hypotheses. Then, the methods section informs the research design as well as data collection and data analysis procedures. Hereafter, results are presented and discussed. Subsequently, findings are discussed in light of previous literature and conclusions are stated. Finally, theoretical contributions and practical implications are presented, and the research limitations are acknowledged.

## 2. Theoretical framework

### 2.1. Job demand-resource in family business

Karasek (1979) proposed the job demand-control model, which states that a psychological work environment is defined by a combination of job demands and job control. This proposal later evolved into the JDCS, which has been widely used to explain variables influencing the stress level of an organization’s employees (Karasek and Theorell, 1990; Baka, 2020). Demerouti et al. (2001) proposed the job demands and resources (JD-R) model as an extension of the JDCS proposal by Karasek (1979). The JDCS and JD-R model considers that job demands are related to workload, time pressure, and role conflicts, among others (Karasek et al., 1998; Van der Doef and Maes, 1999). Support refers to the social integration of the individual in the workplace (Van der Doef and Maes, 1999).

The JD-R model has been shown to be reliable in different settings. Recently Jamal et al. (2021) have reported that task interdependence, workload pressure, professional isolation, and family interference in work relate to exhaustion and stress. They also suggest that work autonomy, flexibility, and the available technology resources have a positive influence on work-life balance and job satisfaction. Karasek and Theorell (1990) found that employees’ most adverse reactions (e.g., burnout) to work conditions relate to higher levels of work demand and low levels of support at workplace. Ellison and Caudill (2020) found that higher job demands were associated with more job stress among prison officers. Pelfrene et al. (2001) concluded that “feeling stressed” was more strongly associated with psychological demands than with freedom of decision or social support.

When these variables are tested in relation to work satisfaction additional support has been found in relation to the model’s ability to explain occupational health at the workplace. Fernemark et al. (2020) demonstrate that social support when working with a digital practice increased physicians’ job satisfaction in the context of telemedicine services. Yeh (2015) found that the positive influences of job resources on job satisfaction were greater than the negative effect of demands on satisfaction in workers from the East Asian region. Jang et al. (2017) evaluated the factors influencing home health care workers’ job satisfaction and found that demands negatively affect, and resources positively affect these employees’ levels of satisfaction.

Despite their interest, these models have hardly been considered in the specific context of family businesses, and no study has been observed that analyzes the impact that COVID-19 has had on them.

**FIGURE 1:**
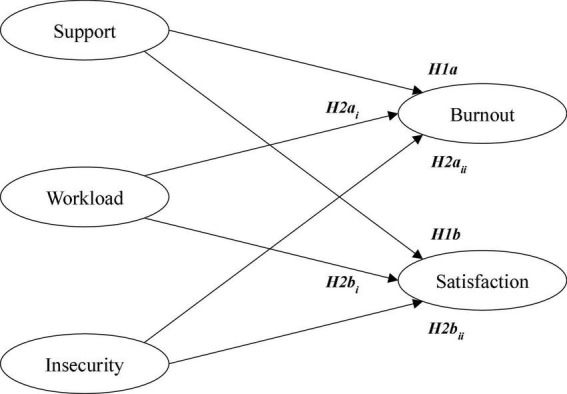
Job demand-resource model in family business.

The JDCS and JD-R model has recently been used to test occupational health in highly stressful work environments, proving again to be reliable (e.g., Prado-Gascó et al., 2020). Accordingly, it is expected that it will also be reliable in assessing burnout and job satisfaction in family businesses which are facing difficulties as a consequence of the pandemic. Family businesses are a particularly interesting setting to assess this model as these organizations are acknowledged for being closely connected to their employees (Cruz et al., 2012; Llanos-Contreras et al., 2019). Accordingly, they would deploy high levels of support to employees. But also, the COVID-19 pandemic put their continuity under risk which would increase the organizational stress as it has financial and socioemotional wealth costs (Llanos-Contreras et al., 2020; Salvato et al., 2020). The above analysis supports the following two hypotheses and the model proposed in the Figure 1.

***H1a.** Resources (support) reduce the level of burnout of employees in family businesses.*

***H1b.** Resources (support) improve job satisfaction of employees in family businesses.*

***H2a.** Demands (workload and job insecurity) will have a positive effect on burnout levels of employees in family businesses.*

***H2b.** Demands (workload and job insecurity) will have a negative effect on job satisfaction levels of employees in family businesses.*

### 2.2. Fear to COVID-19 and job demand-resource model: Crisis management in family firms

With the development of the COVID-19 pandemic, several studies have explored the consequences of this disaster on people’s mental health (e.g., Bohlken et al., 2020; Ornell et al., 2020; Pfefferbaum and North, 2020; Rajkumar, 2020). Previous research has shown that people are heterogeneous in terms of the levels of fear of this pandemic (Fitzpatrick et al., 2020; Harper et al., 2020; Broche-Pérez et al., 2022). These studies have also shown that the level of fear has direct and indirect effects on people’s mental health and occupational health, as well as in their behavior and mood at work.

In relation to the direct effect, this research agrees that fear of COVID-19 has a negative influence on people’s mental health, level of stress, and generally on people’s mood. Thus, Chen and Eyoun (2021) found that fear of COVID-19 is positively associated with job insecurity and emotional exhaustion in restaurant employees. Labrague and Santos (2020) conclude that a higher level of fear of COVID-19 generates lower job satisfaction and greater psychological distress in nurses on the front line of health care. Khattak et al. (2020) proved that fear of COVID-19 has a significant positive impact on the psychological distress and turnover intention of nurses in Pakistan.

Fear of COVID-19 has also proven to have an indirect effect on variables assessing psychological risk. In this way, Gasparro et al. (2020) found that the effect of perceived job insecurity on depressive symptoms in healthcare workers was weaker among employees with low fear of COVID-19. Similarly, Khan et al. (2021) concluded that the effect of perceived job insecurity on people’s mental health was stronger when employees had a greater fear of COVID-19. It suggested that in a work environment, employees’ fear of COVID-19 will enhance the negative effect of job demand on burnout and job satisfaction, while it will weaken the positive effect of job support on these same variables.

Taken together, people have differences in the level of fear of COVID-19, and empirical results in recent research suggest that fear of COVID-19 would have a moderating effect on the relationships proposed by the JD-R model. It supports the following two hypotheses.

***H3a.** Workers’ fear in disaster situations (e.g., COVID-19) will have a moderating effect on the role of resources and demands on burnout of employees in family businesses.*

***H3b.** Workers’ fear in disaster situations (e.g., COVID-19) will have a moderating effect on the role of resources and demands on job satisfaction of employees in family businesses.*

## 3. Research design

### 3.1. Questionnaire

Data were collected through an online questionnaire applied in January 2021. The questionnaire includes questions about variables of interest, namely: burnout, insecurity, satisfaction, support, workload, and fear of COVID-19, as well as demographic data. The Burnout Assessment Tool (BAT) of 22 items representing the four dimensions of burnout: exhaustion, mental distance, emotional impairment, and cognitive impairment was used to assess the level of burnout (Schaufeli and Desart, 2020). The job insecurity scale was adapted from De Witte (2000) to assess this construct. To measure the level of job satisfaction and organizational support, a scale of Eisenberger et al. (1997) was adapted. To measure workload, the UNIPSICO battery was adapted (Gil-Monte, 2016). Finally, the COVID-19 fear measure was an adaptation from a scale proposed by Fitzpatrick et al. (2020). All items have been measured using a 5-point Likert scale.

The sample contains 214 observations of family business employees from Chile surveyed after 10 month the pandemic in this country. The age of the respondents ranges from 22 to 81 years, with an average of 43 (*M* = 43.29; *SD* = 11.94), and the number of men (*n* = 105) and women (*n* = 109) is balanced. Most of the respondents have a technical or professional higher education degree.

### 3.2. Methods

Each of the latent constructs’ psychometric properties was tested, and satisfactory fit measures were obtained for all the reflective models (Table 1). The constructs’ internal consistency was evaluated with Cronbach’s α and composite reliability (CR), finding adequate indicators, except for the construct workload with a Cronbach’s α of 0.666. However, the variable is maintained in the model considering the rest of the satisfactory fit measures of the construct (Hair et al., 2014).

**TABLE 1:** Reliability and validity assessment.

Construct	KMO	AVE	CR	Cronbach’s α	Factor loadings
Burnout	0.907	0.663	0.887	0.830	>0.700***
Exhaustion				0.892	>0.700***
Mental distance				0.800	>0.700***
Emotional impairment				0.880	>0.700***
Cognitive impairment				0.823	>0.700***
Insecurity	0.713	0.722	0.912	0.871	>0.700***
Satisfaction	0.711	0.744	0.897	0.828	>0.700***
Support	0.828	0.689	0.917	0.886	>0.700***
Workload	0.661	0.596	0.815	0.666	>0.700***

CR, composite reliability; AVE, average variance extracted. */**/***Significance level at 0.10/0.05/0.01.

To determine the sample adequacy for factor analysis, the Kaiser-Meyer-Olkin (KMO) test was used. All constructs have a KMO greater than 0.600; moreover, excepting Workload, the KMO is greater than 0.700, which are considered acceptable (Shrestha, 2021). The convergent validity of the reflective models was tested by the factor loadings (above 0.700) and the average variance extracted (AVE) (above 0.500) (Hair et al., 2014). To assess the discriminant validity the heterotrait-monotrait ratio of correlations (HTMT) was used. According to Hair et al. (2016), the discriminant validity has been established between two reflective constructs, if the HTMT value is below 0.900. We tested all pairs of constructs and found no HTMT greater than 0.900, thus, indicators were satisfactory for all cases.

A partial least squares structural equation model (SEM-PLS) was used to test the proposed hypotheses, using burnout and satisfaction levels as dependent variables, organizational support as an explanatory variable of resource, and workload and job insecurity as independent variables of demand. To evaluate the moderating effect of fear of COVID-19, a MGA was used considering three subsamples: individuals with high, medium, and low fear of COVID-19 (Hair et al., 2017a).

## 4. Results

### 4.1. Job demand-control-support in family firms

The multiple correlation coefficient (*R*^2^ = 0.412) and Stone-Geisser’s predictive relevance test (*Q*^2^ = 0.261, blindfolding procedure, omission distance = 7) indicate that the structural model for the dependent variable burnout is relevant and predictive (Chin, 1998, 2010). Also, the standardized root-mean-square residual (SRMR), as a goodness of fit measure, indicates a good fit (SRMR = 0.079) (Henseler et al., 2014). In the model with dependent variable satisfaction, good measurement indicators were also observed (*R*^2^ = 0.415; *Q*^2^ = 0.297, blindfolding procedure, omission distance = 7). It is possible to confirm the significance of both models (Hair et al., 2017b). The SRMR indicates a good fit (SRMR = 0.075) (Henseler et al., 2014).

**FIGURE 2:**
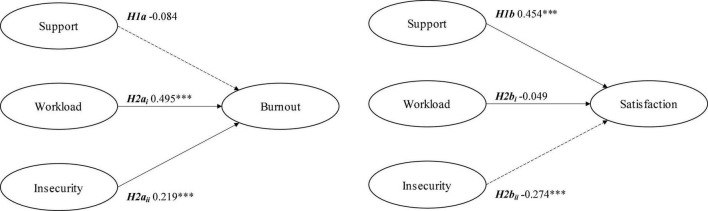
Job demand-resource model in family business. Hypotheses testing.

Support was not found for hypothesis 1a since no significant relationship was found between organizational support and employees’ burnout level in family businesses (Table 2). The analysis results support hypothesis 1b, i.e., organizational support in family businesses improves worker satisfaction in a positive and significant way. Hypothesis 2a is accepted since demands (workload and job insecurity) positively and significantly affect employees’ burnout levels in family businesses. Hypothesis 2b is partially supported since only a negative and significant relationship was found between job insecurity and satisfaction, and no significant effect of workload on job satisfaction was observed. Figure 2 summarizes these informed results.

**TABLE 2:** Results of the SEM-PLS analysis.

	Burnout		Satisfaction		Fit measures	
	**Path**	***f* ^2^**	**Path**	***f* ^2^**	***R* ^2^**	***Q* ^2^**
Support	-0.084	0.009	0.454***	0.275		
Workload	0.495***	0.359	-0.049	0.003		
Insecurity	0.219***	0.062	-0.274***	0.097		
Burnout					0.412	0.261
Satisfaction					0.415	0.297

*/**/***Significance level at 0.10/0.05/0.01; effect sizes (Cohen, 1988): *f*^2^ > 0.35 strong effect; *f*^2^ > 0.15 moderate effect; *f*^2^ > 0.02 weak effect.

### 4.2. Demands and resources model in a pandemic scenario

The MGA shows significant differences among the three proposed groups. Thus hypothesis 3 is supported, confirming that fear of COVID-19 has a moderating effect on the influence that resources and demands have on burnout and job satisfaction. The variable “support” significantly decreases burnout in employees of family firms with high fear of COVID-19. However, it does not affect the satisfaction of workers with a lower level of fear of the disease. Job insecurity presents a positive and significant influence on burnout only for employees with a medium level of fear of COVID-19. It has no effect on job satisfaction in employees of family firms with a low level of fear of COVID-19. The workload effects on burnout level and job satisfaction show similar results to the overall model, but the effect of workload on burnout is highest for employees with low fear of COVID-19. Table 3 presents all these results.

**TABLE 3:** Results of the multigroup analysis.

	Low fear			Medium fear			High fear		
		**Path**	***f* ^2^**		**Path**	***f* ^2^**		**Path**	***f* ^2^**
Support → burnout	–	0.247*	0.119		0.047	0.007	–	0.233**	0.073
Workload → burnout		0.675***	1.075		0.450***	0.264		0.496***	0.408
Insecurity → burnout		0.000	0.000		0.347***	0.154		0.150	0.030
R^2^ burnout		0.652			0.409			0.434	
Support → satisfaction		0.249	0.067		0.590***	0.528		0.396***	0.216
Workload → satisfaction	–	0.257	0.086		0.018	0.000	–	0.024	0.001
Insecurity → satisfaction	–	0.278	0.089	–	0.218**	0.070	–	0.373***	0.189
R^2^ satisfaction		0.372			0.490			0.446	

*/**/***Significance level at 0.10/0.05/0.01; effect sizes (Cohen, 1988): *f*^2^ > 0.35 strong effect; *f*^2^ > 0.15 moderate effect; *f*^2^ > 0.02 weak effect.

## 5. Discussion and conclusion

It is acknowledged in family firm literature that for these organizations, continuity and survival (transgenerational) are one of their main goals (Kotlar and De Massis, 2013; Llanos-Contreras and Jabri, 2021), and COVID-19 had been a major shock which has threatened this aim in recent times. One way through which the pandemic challenged these firms’ continuity was by the impact it has had on their human resources’ psychological risk, due to psychological risk impact on individuals’ health, organizational climate, and firm productivity (Bergh et al., 2018). Psychological risk can arise as consequence of poor social context, work, and organizational design, and COVID-19 is considered to have had a great negative impact on these (Prado-Gascó et al., 2020). Importantly, burnout, and job satisfaction are related to psychological risk at work (Guadix et al., 2015; Elshaer et al., 2018). For this reason, this article focuses on responding to the questions: how do demands and resources variables affect the level of burnout and satisfaction of employees of family businesses in the context of the pandemic (COVID-19)? How does fear of COVID-19 affect the influence of demands and resource variables on the level of burnout and employee satisfaction in family firms?

In relation to job demands, this study’s results confirm the theoretical predictions that workload and insecurity positively influence burnout, as well as confirming that job insecurity negatively influences job satisfaction. This is in line with previous studies on psychological risk at work (e.g., Elshaer et al., 2018; Ellison and Caudill, 2020; Jamal et al., 2021). Job demands produce important negative effects on occupational health in firms facing stressful scenarios. Family firms need to be careful about how they manage these two elements particularly when facing stressful scenarios like the one imposed by COVID-19. While adapting operations to new conditions and challenges imposed by the external shock will increase workload, the highly acknowledged family commitment with business continuity can be a positive signal to enhance job stability (Gómez-Mejía et al., 2007; Llanos-Contreras et al., 2019). Thus, family firms, particularly those small and medium size ones which have fewer resources to adjust to the changes required by an external shock, would be able to counterbalance the additional demand of workload with clear signals of commitment to business continuity and job stability.

In relation to job resources, results provide empirical support to the idea that job support positively influences job satisfaction. Similar to the prediction on job demands, these results reinforce previous literature in relation to the importance of this for an organization to keep psychological risk under control (Yeh, 2015; Jang et al., 2017; Fernemark et al., 2020). Karasek (1979)’s model proposes that occupational health problems arise as a consequence of the imbalance between psychological demands and the resources workers have available to manage such demands. Job support is a critical resource for keeping psychological demand and resources in balance and providing good occupational health. Job support is related to the concern the organization sets on the workers’ welfare, their availability to provide help when the workers have a problem, the firm’s disposition to consider the workers’ opinion and/or help in solving problems if the worker were to make any mistake (Eisenberger et al., 1997; Zeng et al., 2020). In line with Prado-Gascó et al. (2020)’s results, this study suggests that job support was important for people at work to overcome the stress, uncertainty, and pressure consequences of the pandemic scenario.

In relation to how fear of COVID-19 affects the influence of demands and resources on burnout and job satisfaction in family firms, overall, the proposed moderating effect is confirmed. It is interesting to see that despite the fact that the whole sample analysis did not provide empirical support to the predicted negative influence of support on burnout, this relationship is confirmed when responses from people with high levels of fear of COVID-19 are analyzed. Similarly, a moderating effect of fear of COVID-19 is also observed for the relationships between insecurity and burnout, support and satisfaction, as well as for the relationship between insecurity and satisfaction. These results are in line with recent research indicating that people are heterogeneous in terms of their level of fear of COVID-19 and that this heterogeneity is important in people’s mental health assessment (Gasparro et al., 2020; Harper et al., 2020; Labrague and Santos, 2020; Chen and Eyoun, 2021; Broche-Pérez et al., 2022). This is important for organization management and family firm theory as managers and family business owners need to consider such heterogeneity in fear of COVID-19 when making decisions that affect workload, insecurity, and job support at work. Family business owners and managers need to be particularly concerned about those workers who present higher levels of fear of COVID-19, as they will be particularly sensitive to be negatively affected by actions and situations increasing job demands and less likely to positively respond to job support policies.

Overall, the results discussed in the previous section confirm the JR-D model predictions for family business workers facing the shock of the COVID-19 pandemic (Karasek, 1979; Demerouti et al., 2001). This is important because it proves the reliability of the model even under conditions of extreme external pressure. Data were collected when the pandemic was at its peak in Chile, before the vaccine was found and under conditions of high uncertainty about how long it would last and what consequences it would have for people, small-medium family businesses, and the economy. Thus, support is given to the idea that family firms facing stressful scenarios can control psychological risk at work by managing job demand and resources in order to enhance continuity and survive. Literature on family businesses shows these organizations as highly resilient and suggests that owners-managers look to continue operating despite minimal financial reward and highly adverse conditions (Glover and Reay, 2015; Llanos-Contreras et al., 2020; Mahto et al., 2022). However, in this work, it is suggested that keeping workers’ occupational health under control is central to meet this aim, and for this family, firms need to be aware of the effect of their decisions on JD-R.

### 5.1. Contributions to theory and practice

This study makes contributions for theory and practice. In terms of theoretical contributions: First, contributions are made to the literature on how family firms face severe external shocks and survive (Marshall and Schrank, 2020; Mahto et al., 2022). Family firms are particularly acknowledged for their priority of preserving the firm through generations which leads them to do everything within their reach to overcome difficulties and survive. Such resilience has been related to the priority for preserving the socioemotional wealth the firm provides to the family. However, continuing business operation is not just about motivation for preserving socioemotional wealth, but also about these firm’s ability to manage shock and adapt to changing contextual conditions (Alonso-Dos-Santos and Llanos-Contreras, 2019; Salvato et al., 2020). In this work we suggest that managing occupational health is an important factor. Thus, the psychological resources and demands these firms should consider managing when their workers face stressful scenarios are analyzed. Second, this study also contributes to the literature on organizational psychology (Bennis et al., 1966; Beehr, 1998), particularly to the study of factors influencing psychosocial risk. The study first applied the acknowledged JD-R model to determine how demand and resource factors influenced psychological risk in family firms when faced with the COVID-19 pandemic. In this way, the importance of balancing workload, job insecurity, and job support to keep occupational health under control is discussed.

The study also shows the importance of observing workers’ heterogeneity in terms of fear of COVID-19 as it is a driver which can increase or decrease the impact of the resources and demand variables on psychological risk. Finally, this study contributes to the very scant research on Latin American family firms, a region where these organizations are particularly prevalent (Gomez-Mejia et al., 2020; Vazquez et al., 2020). Chile was acknowledged for its efficiency in managing the crises generated by COVID-19 from a public health point of view. However, it imposed severe restrictions on economic activities and high demands on companies to continue to operate under pandemic conditions. This implies pressure and stress not only for family business owners whose income is reduced and face additional expenses for adapting their operation but also for workers who see their occupational health and job at risk and face changes in the way they do their jobs.

This article also has important practical implications. Family business owners and managers can benefit by learning how they should manage workload, send the correct signals about business continuity to enhance workers’ perception of job stability, and provide enough job support in order to keep psychological risk at work under control. This is not only important for business policy but also for public policy, since government authorities have much to do in terms of providing the correct information and certainty about what these firms need to do to continue operations in a safe way. This will be important for the business’ operation but also for their workers’ occupational health. Practitioners can also learn about the importance of considering the asymmetries on the level of fear of COVID-19 that people present. This is important in order to develop differentiated policies that address the specific needs of groups of people with different levels of risk of presenting occupational health problems.

### 5.2. Limitations and future research

This research has some limitations that can be considered as a starting point for future work. The development of the pandemic demonstrated that countries have very different capacities and resources to deal with this type of event. It is possible that the best prepared countries will be able to maintain an economic flow that will allow companies to survive in these uncertain contexts. Thus, the economic situation of the country during the COVID-19 pandemic may influence workers’ perceptions and their fear of the pandemic. Future research can use samples from different countries to contrast results, for example, between developed and emerging economies. The findings of this study can even be analyzed among autonomous states belonging to the same country to understand the differences within a decentralized governmental system.

This study has not considered how family firms monitor and cope with employees’ fear of disastrous events (e.g., COVID-19 pandemic). It would be interesting to study the behavior of family firms in terms of the mechanisms they use to manage employees’ negative perceptions, insecurities, and fear, which can compromise the survival of the firm. Moreover, the way family businesses act toward their employees in times of crisis can influence the reputation of the firm and threaten one of the main competitive advantages of the family business. In this vein, an avenue of research can turn its attention to the strategies that family firms implement for managing human resources during disasters (e.g., COVID-19 pandemic) and how these strategies affect the reputation of the company.

Considering the heterogeneity of family businesses, some of them may be better prepared than others to face the pandemic or may be in sectors that have not been negatively affected by this event. Analyzing the differences in the capabilities of family businesses to cope with the pandemic may serve as an example for other family businesses to adopt successful strategies to survive during adverse events.

## Data availability statement

The raw data supporting the conclusions of this article will be made available by the authors, without undue reservation.

## Author contributions

All authors listed have made a substantial, direct, and intellectual contribution to the work, and approved it for publication.
